# Exploring the reach and program use of hello world, an email-based health promotion program for pregnant women in the Netherlands

**DOI:** 10.1186/1756-0500-5-514

**Published:** 2012-09-22

**Authors:** Johanna M van Dongen, Mireille NM van Poppel, Ivon EJ Milder, Hans AM van Oers, Johannes Brug

**Affiliations:** 1 Department of Public and Occupational Health and the EMGO + Institute for Health and Care Research, VU University Medical Center, van der Boechorststraat 7, Amsterdam, 1081 BT, the Netherlands; 2 Department of Health Sciences and the EMGO + Institute for Health and Care Research, Faculty of Earth and Life Sciences, VU University Amsterdam, de Boelelaan 1085, Amsterdam, 1081 HV, the Netherlands; 3 Centre for Prevention and Health Services Research, National Institute for Public Health and the Environment, Antonie van Leeuwenhoeklaan 9, Bilthoven, 3721 MA, the Netherlands; 4 Centre of Public Health Forecasting, National Institute of Public Health and the Environment, Antonie van Leeuwenhoeklaan 9, Bilthoven, 3721 MA, the Netherlands; 5 Scientific Center for Care and Welfare, AB Tilburg University, Warandelaan 2, Tilburg, 5037, the Netherlands; 6 Department of Epidemiology and Biostatistics and the EMGO + Institute for Health and Care Research, VU University Medical Center, Van der Boechorststraat 7, Amsterdam, 1081 BT, the Netherlands

**Keywords:** Pregnancy, Health promotion, Internet, Reach, Program engagement

## Abstract

**Background:**

In 2006, the Dutch government initiated Hello World, an email-based program promoting healthy lifestyles among pregnant women through quizzes with pregnancy-related questions. In 2008, an updated version was released.

The present study aimed to (1) examine the reach of Hello World and the representativeness of its users for all pregnant women in the Netherlands, (2) explore the relationship between program engagement and lifestyle characteristics, and (3) explore the relationship between the program content participants accessed (content on smoking, physical activity, and nutrition) and their lifestyle characteristics.

**Methods:**

Data from 4,363 pregnant women were included. After registration, women received an online questionnaire with demographic and lifestyle questions. To evaluate their representativeness, their demographic characteristics were compared with existing data for Dutch (pregnant) women. Women were classified on the following lifestyle characteristics: smoking, nutrition, physical activity, and pre-pregnancy weight status. Program use was tracked and the relationships between lifestyle characteristics, program engagement, and the percentage of smoking, physical activity, and nutrition questions accessed after opening a quiz were explored using Mann–Whitney U tests and Kruskal-Wallis tests.

**Results:**

Hello World reached ±4% of its target population. Ten percent of participants were low educated and 22% immigrants. On average, women received 6.1 (SD:2.8) quiz emails and opened 32% of the associated quizzes (2.0, SD:2.1). A significant positive association was found between the number of quizzes opened and the number of healthy lifestyle characteristics. After opening a quiz, women accessed most smoking, nutrition, and physical activity questions. Significant relationships were found between several lifestyle characteristics and the percentage of smoking, physical activity, and nutrition questions accessed. However, between-group differences were small, quiz topics were largely unrelated to their lifestyle characteristics, and inconsistencies were found regarding the directions of these associations.

**Conclusions:**

Hello World reached ±4% of its target population, which is lower than the reach of its previous version (±8%). Relatively few low educated and immigrant women registered for the program. Active participation in the program was positively associated with the number of healthy behaviours participants engaged in. The program content participants chose to access was largely unrelated to their lifestyle characteristics.

## Background

Smoking, unhealthy dietary habits, and an unhealthy weight status during pregnancy may negatively influence maternal health, pregnancy course, and pregnancy outcomes [1-6]. Despite the negative consequences, these unhealthy lifestyle characteristics are prevalent among pregnant women. Approximately 17% of Dutch women smoke during their pregnancy [7] and a recent Dutch national food-consumption survey indicated that pregnant women eat too little fruit and vegetables (i.e. on average 110 grams of fruit and 122 grams of vegetables a day despite the Dutch Health Council recommendation of a minimum daily intake of two servings of fruit and 200 grams of vegetables [8,9]). Furthermore, approximately 23% of Dutch women of childbearing age (15–45 years of age) are overweight (25 kg/m^2^ ≤ [BMI (Body Mass Index)] <30 kg/m^2^), 10% are obese ([BMI] ≥30 kg/m^2^), and about 50% do not meet the Dutch recommendation for physical activity [10].

Since most pregnant women welcome health-related information and actively search for it on the Internet [11], the Internet provides a useful setting for interventions aimed at promoting healthy behaviours during pregnancy. Furthermore, online health information has the potential to reach large audiences at relative low costs [12], and the Internet allows participants to access an intervention when and where they want, and in a relatively anonymous manner [13]. Another advantage of the Internet is that it provides the opportunity to offer interactive, individualized interventions that can be matched to the visitor’s characteristics [14].

In 2006, the Dutch government initiated Hello World [*Hallo Wereld* in Dutch], an email- and web-based program promoting healthy behaviours among pregnant women. Hello World provides generic information about various lifestyle topics (e.g. smoking, nutrition, and physical activity), but its core element is a series of quizzes with pregnancy-related lifestyle questions (and answers) tailored to the number of gestational weeks. The first version of the program was pilot-tested in Amsterdam, and then improved and implemented nationwide [11,15]. During the first year after implementation, approximately 8% of Dutch pregnant women enrolled in the program; however, immigrants and women with a low level of education were underrepresented [15]. In 2008, an updated version was released.

Previous research indicates that online interventions may be effective in motivating people to adopt healthy behaviours [16,17]. However, for a nationwide program like Hello World to have a substantial impact on the general health of the population, it is important that it reaches a sizeable proportion of its target population. In the “Integrated Model for exploring motivational and behaviour change” (I-Change; [18]), awareness about one’s risk behaviour(s) is regarded as a necessary prerequisite for health behaviour change and knowledge is seen as an important factor in accomplishing this [18]. Therefore, Hello World should not only reach its target population, but it’s participants should also be actively engaged in the program to acquire as much knowledge as possible and at-risk participants should be exposed to the program content concerning their risk behaviour(s). In the case of Hello World, for example, information about the adverse effects of smoking during pregnancy is of particular importance for women who (intend to) smoke during their pregnancy.

Previous research indicates that program reach and engagement are often not optimal in web-based interventions [14] and that both are related to the characteristics of the intervention and participants (e.g. lifestyle characteristics) [12,14,19]. To our knowledge, studies exploring the relationship between the lifestyle characteristics of participants and the program content that they chose to access are lacking. The latter is of particular importance for programs like Hello World, since they aim to achieve health behaviour change by simultaneously providing information on various lifestyle topics. Therefore, the aim of the present study was threefold:

1. To examine the reach of Hello World and the representativeness of its users for all pregnant women in the Netherlands. Reach was defined as the proportion of the intended target population (i.e. Dutch pregnant women) that participated in the intervention [20].

2. To explore the relationship between program engagement and the lifestyle characteristics of participants. Program engagement was explored in terms of dose received and dose delivered. Dose received was defined as the number of intended program units delivered (i.e. number of quiz emails received) and dose delivered as the extent to which participants actively engaged and interacted with the program content (i.e. number of quizzes opened) [20].

3. To explore the relationship between the program content that participants chose to access (i.e. quiz questions on smoking, physical activity, and nutrition) and their lifestyle characteristics (i.e. smoking, nutrition, physical activity, and pre-pregnancy weight status). Since pregnant women may actively search for health information about their risk behaviour(s) after registering for Hello World, it was hypothesized that the following were positively related: the percentage of smoking questions accessed after opening a quiz and unhealthy characteristics for smoking status, the percentage of physical activity questions accessed after opening a quiz and unhealthy characteristics for weight status and physical activity, and the percentage of nutrition questions accessed after opening a quiz and unhealthy characteristics for weight status and nutrition.

## Methods

### Recruitment of participants

Since pregnant women frequently turn to their health care providers for information about what they should and should not do during their pregnancies [21], participants were recruited for Hello World through midwifery practices and gynaecologists. Leaflets were handed out by these professionals and/or so-called ‘Hello World Newsletters’ were distributed in their waiting rooms. Leaflets were also included in so-called ‘pregnancy gift boxes’, i.e. boxes with a variety of pregnancy-related presents provided by commercial organisations that can be (and very often are) requested by pregnant women and sent to their home address. Banners were placed on several pregnancy-related websites, and advertisements for Hello World appeared when performing pregnancy-related searches in Google. Furthermore, since a previous program evaluation had indicated that immigrants were underrepresented [15], special efforts were made to reach them by placing ads on various minority group websites. As Hello World was freely available online, everyone with Internet access who was aware of the program could register.

### Inclusion of participants

Pregnant women who registered for Hello World between March 25, 2009 and October 25, 2009 were included in the study. No specific exclusion criteria were stated, but several user accounts were excluded from the analyses as they were regarded as errors.

Upon registration, participants were asked to enter their first name, date of birth, email address, postal code, expected date of delivery, education level, place of birth, and that of their parents. Directly after registration, the women received an invitation to complete an online questionnaire with 44 items assessing their demographic and lifestyle characteristics (completion time: 15–20 minutes). It was explained to the participants that answering this questionnaire was voluntary and not a prerequisite for (further) use of the website [15].

Accounts of test users (n = 6), non-pregnant users (n = 97), and those who filled in two distinct dates of birth upon registration and while answering the online questionnaire were excluded (n = 2). Assuming a pregnancy duration of 40 weeks, the number of gestational weeks at registration was calculated based on the date of program registration and the expected delivery date. Users whose entries indicated an unlikely pregnancy duration (less than 0 or more than 42 weeks) (n = 159) were excluded. Furthermore, several participants registered more than once. In those cases, the least active account was excluded (n = 79). Age was calculated by date of birth. Women who noted ages of less than 15 and more than 45 years were regarded as errors and were therefore excluded (n = 16). Eventually, data from 4,363 women were included.

Ethical approval was not necessary for the present study, since it is not required for studies that do not affect participants’ integrity (according to the Dutch Medical Research Involving Human Subjects Act (WMO)). The study was carried out in accordance with Dutch privacy legislation. All participants gave informed consent by agreeing to the user conditions of the website when registering.

### Intervention

**Figure 1:**
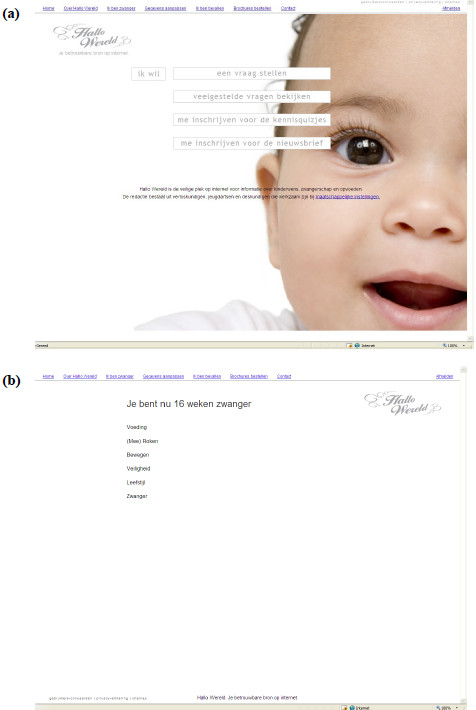
**Lay-out of the Hello World website.** (**a**) Main page, (**b**) Quiz questions.

Hello World was launched on November 13, 2006 and was aimed at promoting healthy behaviours among pregnant women by providing information about various lifestyle topics. The program was developed in association with several Dutch health promotion institutes (e.g. The Netherlands Institute for Sports and Physical activity, and The Netherlands Nutrition Centre) and the Dutch Organisation of Midwives. An interactive approach was chosen for delivering the health information (i.e. quiz emails), since this is thought to promote active information processing and user satisfaction [22]. On the program website, women could register for free at anytime during their pregnancy. After registration, they received quizzes containing pregnancy-related lifestyle questions applicable to their stage of gestation. The first quiz email was sent at 8 weeks of pregnancy and subsequent quiz emails were sent every four weeks until delivery. Participants were informed about new quizzes by emails with an example of the quiz questions and a hyperlink to the entire quiz. Quizzes had a maximum of seven questions including one on each of the following lifestyle topics: smoking, nutrition, physical activity, lifestyle/care, pregnancy, safety, and emotions. Participants were free to answer as many questions as they wanted. Each question had two possible answers. After an answer was selected, it was scored (i.e. correct or incorrect) and an explanation of the correct answer was provided. Most answers included a practical tip for behaviour change and a hyperlink to the website of the related health promotion institute. On the program website, women could also ask pregnancy-related questions of experts of Dutch health promotion institutes and the Dutch Organisation of Midwives. To reach women with a low level of education, the health information was presented in plain language and short text blocks [11,15]. In contrast to the previous version, the program included an additional weekly newsletter, a new layout (Figure 1), and seven instead of six questions per quiz. The newsletter was added because a previous program evaluation indicated that the main suggestion for improvement among program users was to expand the amount of health information [23].

### Measures

#### Demographic characteristics

Education level was assessed as the highest level of education a woman had completed and was categorized as low (i.e. lower secondary education or less), intermediate (i.e. higher secondary education), or high (i.e. college or university).

Ethnicity was defined according to the definition(s) of Statistics Netherlands [24]. Participants born outside the Netherlands were considered first-generation immigrants. If participants were born in the Netherlands, but at least one of their parents was born outside the Netherlands, they were considered second-generation immigrants. Based on their country of birth, immigrants were also divided into groups from Western- and non-Western countries.

#### Lifestyle characteristics

Participants completing the online questionnaire were classified on the following lifestyle characteristics: smoking, nutrition (i.e. fruit and vegetable intake, and eating breakfast), physical activity, and pre-pregnancy weight status.

Smoking status was assessed using two items developed for the previous program evaluation [15]. Participants were asked whether they currently smoked cigarettes (No, never; No, I quit a long time ago; No, I quit because of the pregnancy; Yes, but I intend to quit; Yes). They were also asked whether they had been exposed to tobacco smoke in their own home during the past 7 days (No, nobody ever smokes in my home; No, never in my presence; No, not during the past 7 days; Yes). Participants who reported that they currently smoked cigarettes were classified as “smokers”, participants who reported that they did not smoke themselves, but who were exposed to tobacco smoke in their own home during the past 7 days, were classified as “passive smokers”, and all others as “non-smokers”.

Fruit and vegetable intake was assessed using four items of a Community Health Services food-consumption questionnaire [25]. Participants were asked to report their regular fruit intake in pieces of fruit per day and their average daily number of serving spoons of vegetables (i.e. one serving spoon was defined as 50 grams). The Dutch Health Council recommends an average daily intake of at least two servings of fruit and 200 grams of vegetables [8]. Participants who met the Dutch Health Council criteria were classified as “eating sufficient fruit and vegetables”, and all others were classified as “eating insufficient fruit and vegetables”.

Eating breakfast was assessed using one item of a Community Health Services food-consumption questionnaire [25]. Participants were asked to report the average number of days per week that they eat breakfast. Participants who ate breakfast at least five times a week were classified as “regularly eating breakfast”, and all others as “irregularly eating breakfast”.

Physical activity was assessed using a single question asking participants whether they participated in moderate-intensity physical activities for a cumulative minimum of 30 minutes a day and for five or more days a week (i.e. the Dutch recommendation for physical activity [8]). Participants meeting this recommendation were classified as “physically active”, and all others as “physically inactive”.

Pre-pregnancy weight status, based on self-reported pre-pregnancy weight and height, was calculated per participant as body mass index (BMI; calculated as weight in kilograms divided by the square of height in meters). According to their pre-pregnancy weight status, participants were classified as “underweight” (BMI < 18,5 kg/m^2^), “normal weight” (18,5 ≤ BMI < 25 kg/m^2^), “overweight” (25 ≤ BMI < 30 kg/m^2^), or “obese” (BMI ≥ 30 kg/m^2^).

The number of healthy lifestyle characteristics (range: 0–5) was calculated per participant by summing their total number of healthy lifestyle characteristics (i.e. non-smoking, sufficient fruit and vegetable intake, regularly eating breakfast, physically active and normal weight).

#### Program use and program engagement

Program use was registered continuously and included registration data, quiz emails sent, and quiz questions accessed. Upon registration, a referral ID was assigned to each participant, which was integrated in the quiz emails to track their use of the quiz questions. Quizzes were regarded as opened if at least one quiz questions was accessed. To cover a whole pregnancy period, user data were extracted until nine months after the last opportunity to complete the online questionnaire (July 25^th^, 2010). Program engagement was examined in terms of dose received (i.e. number of quiz emails received), dose delivered (i.e. number of quizzes opened), and percentage of received quizzes opened (i.e. dose delivered/dose received) [20]. Furthermore, the percentage of smoking, physical activity, and nutrition questions accessed after opening a quiz was calculated as a ratio of the number of quiz questions accessed versus dose delivered.

### Data analysis

First, the reach of Hello World and the representativeness of its users for all pregnant women in the Netherlands were examined. Program reach was estimated by dividing the number of pregnant women who registered for the program by the expected number of newborns and stillbirths (i.e. foetal death after 24 weeks of gestation) in the Netherlands during the 7-month study period. Demographic characteristics of program users were described, including their number of gestational weeks at registration, age, education level, and ethnicity. To evaluate their representativeness for all pregnant women in the Netherlands, these characteristics were compared with existing data of Dutch pregnant women, and if unavailable, with data of women of childbearing age [26]. Demographic characteristics were also compared between completers and non-completers of the online questionnaire using T-tests and Chi-square tests.

Second, the relationship between program engagement (i.e. dose received, dose delivered, and percentage of received quizzes opened) and lifestyle characteristics of participants was assessed using Mann–Whitney U tests and Kruskal-Wallis tests.

Third, data were analysed to determine whether the program content that participants chose to access was related to their lifestyle characteristics. Therefore, relationships between the percentage of smoking, physical activity, and nutrition questions accessed after opening a quiz and the lifestyle characteristics of participants were assessed using Mann–Whitney U tests and Kruskal-Wallis tests. The percentage of smoking, physical activity, and nutrition questions accessed after opening a quiz were calculated as a ratio of the number of quiz questions accessed versus the dose delivered (i.e. number of quizzes opened). Thus, the results would have been highly influenced by women who opened only a few quizzes. For example, if a woman opened only one quiz and subsequently accessed all associated quiz questions, this would have resulted in rates of 100%. If this same woman had not accessed any of the associated quiz questions, this would have resulted in rates of 0%. Therefore, to obtain more stable and representative results, only data of women who opened 50% or more of the received quizzes were used for these analyses. As the average number of quiz emails received was 6.3 (SD:2.7) among questionnaire completers, the cut-off point was set at three opened quizzes.

T-tests and Chi-square tests were used for normally distributed data and Mann–Whitney U tests and Kruskal-Wallis tests for non-normally distributed data. Data were analysed using SPSS 15 with a level of significance of p < 0.05.

## Results

### Program reach and representativeness of participants

Based on the number of pregnant women who registered for the program during the 7-month study period (n = 4,363) and the number of living newborns and stillbirths in the Netherlands during a 7-month period in 2009 (n = 108,245) [26], it was estimated that approximately 4% of all Dutch pregnant women registered for Hello World (Table 1). Relatively few women with a low level of education (398/4363, 10%) registered for the program compared to the proportion found in Dutch women of childbearing age (36%) [26]. The percentage of immigrants (915/4,363, 22%), first-generation immigrants (425/4,363, 12%), and non-Western immigrants (628/4,363, 15%) among participants were also lower than those found in Dutch women of childbearing age [26].

**Table 1:** Demographic characteristics of Hello World participants and pregnant women in the Netherlands

	**Hello World**						**the Netherlands**^**b**^
	**All**		**Completers**^**a**^		**Non-completers**		
	**n**		**n**		**n**		
**Number of pregnant women**	4363	-	1369	-	2994	-	108245
**Number of weeks pregnant at registration (Mean ± SD)**	4363	15 ± 10	1369	14 ± 9*	2994	16 ± 10*	-
**Age at due date (Mean ± SD)**	4351	30 ± 5	1367	30 ± 5	2984	30 ± 5	31^d^
**15-25y (%)**	616	14	180	13	436	15	10^e^
**25-35y (%)**	2925	67	939	67	1986	67	65^e^
**35-45y (%)**	808	19	247	18	561	19	25^e^
**Education level**	4087	-	1323	-	2764	-	
**Low (%)**	398	10	134	10	264	10	36^f^
**Intermediate (%)**	1714	42	564	43	1150	42	44^f^
**High (%)**	1978	48	625	47	1350	49	20^f^
**Ethnicity**	4291	-	1369	-	2922	-	
**Immigrant (%)**	915	22	253	19*	662	23*	27^e^
**First generation immigrant (%)**	425	12	104	8*	321	11*	17^e^
**Non-Western immigrant (%)**	628	15	153	11*	475	16*	17^e^

Abbreviations; SD: Standard Deviation, n: number, y: years.

* = Significant at p < 0.05.

^a^ Online questionnaire completers.

^b^ Data from Statistics Netherlands [26].

^c^ Number of living newborns and stillbirths in the Netherlands during a 7-month period in 2009.

^d^ Mean age at due date of all pregnant women in the Netherlands in 2009.

^e^ Data from pregnant women only.

^f^ Data from all women aged from 15–45 years.

### Lifestyle characteristics of participants

The online questionnaire was completed by 1,369 of the 4,363 women who registered for the program during the study period (response rate: 31%). Completers and non-completers were comparable except for their number of weeks of gestation at registration and their ethnicity (Table 1). The average pregnancy duration of the completers was approximately two weeks longer (M = 16.0, SD = 10.1) than that of the non-completers (M = 14.1, SD = 8.8), t(4361) = 6.0, p = .00. Fewer immigrants (253/1369, 19%) completed the online questionnaire, X^2^(1, N = 4291) = 9.69, p = .002. During their pregnancy, approximately 30% (405/1355) of participants reported to comply to the Dutch recommendation for physical activity, 13% (150/1127) to the Dutch fruit and vegetable recommendation, 88% (1196/1363) ate breakfast on a regular basis, 12% (158/1359) were active smokers, and 13% (174/1359) were passive smokers. Based on their pre-pregnancy weight status, 4% (47/1190) were classified as underweight, 22% (258/1190) as overweight, and 11% (136/1190) as obese (Table 2).

**Table 2:** Dose received, dose delivered, and percentage of received quizzes opened versus lifestyle characteristics of participants

	**Number of participants (n {%})**	**Dose received**^**b**^**(Mean ± SD)**	**Dose delivered**^**c**^**(Mean ± SD)**	**Received quizzes opened (%) (Mean ± SD)**	**≥3 quizzes opened (%)**
**All**	4363				
Completers^**a**^	1369{31}	6.3 ± 2.7*	2.5 ± 2.3*	40 ± 33*	40
Non-completers	2994{69}	6.0 ± 2.8*	1.7 ± 2.0*	29 ± 30*	26
**Lifestyle characteristics**					
**Physical Activity**	1355				
Active	405{30}	6.1 ± 2.8	2.5 ± 2.4	40 ± 34	41
Inactive	950{70}	6.3 ± 2.7	2.6 ± 2.4	41 ± 33	61
**Nutrition *(Fruit and Vegetables)***	1127				
Sufficient	150{13}	6.5 ± 2.6	3.0 ± 2.5*	49 ± 34*	47
Insufficient	977{87}	6.2 ± 2.7	2.5 ± 2.4*	40 ± 36*	40
**Nutrition *(Breakfast)***	1363				
Regular	1196{88}	6.3 ± 2.7	2.5 ± 2.4	41 ± 34	40
Irregular	167{12}	6.2 ± 2.9	2.3 ± 2.3	36 ± 32	38
**Smoking**	1359				
Active smoking	158{12}	6.0 ± 3.0	2.0 ± 2.2*	31 ± 30*	31
Passive smoking	174{13}	6.0 ± 2.9	2.2 ± 2.3*	37 ± 33*	36
Non-smoking	1027{75}	6.4 ± 2.6	2.7 ± 2.4*	42 ± 34*	42
**Body Mass Index**	1190				
Underweight	47{4}	5.8 ± 2.8	1.7 ± 1.9*	31 ± 33	26
Normal weight	749{63}	6.6 ± 2.6	2.7 ± 2.4*	41 ± 33	43
Overweight	258{22}	6.3 ± 2.7	2.5 ± 2.3*	39 ± 34	39
Obese	136{11}	5.8 ± 2.9	2.6 ± 2.6*	46 ± 37	38
**Number of healthy lifestyle characteristics**	973				
0	13{1}	5.7 ± 3.0	3.2 ± 2.4*	43 ± 36*	54
1	94{10}	6.0 ± 2.9	1.9 ± 2.3*	30 ± 32*	33
2	280{29}	6.2 ± 2.7	2.5 ± 2.4*	40 ± 34*	39
3	368{38}	6.5 ± 2.6	2.6 ± 2.3*	40 ± 33*	42
4	188{19}	6.2 ± 2.6	2.9 ± 2.5*	47 ± 36*	46
5	30{3}	6.3 ± 3.0	3.4 ± 2.7*	56 ± 35*	53

Abbreviations; n: number, SD: Standard Deviation.

^a^ Online questionnaire completers.

^b^ Number of quiz emails received.

^c^ Number of quizzes opened.

* Significant at p < 0.05.

### Dose received and dose delivered versus lifestyle characteristics of participants

On average, women received 6.1 (SD = 2.8) quiz emails and responded to 32% of them by opening the associated quiz (M = 2.0, SD = 2.1). Women who completed the online questionnaire had received 6.3 (SD = 2.7) quiz emails and responded to 40% (M = 2.5; SD = 2.3). Women who did not non-complete the online questionnaire had received 6.0 (SD = 2.8) quiz emails and responded to 29% (M = 1.7; SD = 2.0). Participants with healthy and unhealthy lifestyle habits for physical activity, fruit and vegetable intake, eating breakfast, smoking, and pre-pregnancy weight status did not differ significantly in the number of quiz emails received. Women who reported to eat sufficient amounts of fruit and vegetables (Mann Whitney U test; U = 59908, p = .00) and non-smokers (Kruskal-Wallis Chi-Square = 18.9, p = .00) opened significantly more of the received quizzes than women with unhealthy habits for these lifestyle characteristics respectively. Furthermore, a significant positive association was found between the number of received quizzes opened and the number of healthy lifestyle characteristics (Kruskal-Wallis Chi-Square = 14.7, p = .01) (Table 2).

### Accessed program content versus lifestyle characteristics of participants

After opening a quiz, questionnaire completers accessed on average 84% of smoking questions, 88% of nutrition questions, and 88% of physical activity questions (Table 3). Physical activity, smoking status, pre-pregnancy weight status, and the number of healthy lifestyle characteristics were not significantly related to the percentage of physical activity, smoking, and nutrition questions accessed after opening a quiz. Fruit and vegetable intake was not significantly related to the percentage of quiz questions accessed about smoking and nutrition. However, after opening a quiz, women who reported to eat insufficient amounts of fruit and vegetables accessed significantly less questions about physical activity compared to women who ate sufficient amounts of fruit and vegetables (Mann Whitney U = 12091.0, p = .03). Women who did not eat breakfast on a regular basis accessed significantly more questions about smoking and nutrition than their healthy counterparts (smoking: Mann Whitney U test; U = 12647.0, p = .01, breakfast: Mann Whitney U test; U = 13179.5, p = .03). The percentage of physical activity questions accessed was not related with eating breakfast.

**Table 3:** Percentage of smoking, nutrition and physical activity questions accessed after opening a quiz versus participants’ lifestyle characteristics

	**Number of participants (n)**	**Smoking questions accessed (% ± SD)**	**Nutrition questions accessed (% ± SD)**	**Physical activity questions accessed (% ± SD)**
**All**	1326			
Completers^**a**^	549	84 ± 24*	88 ± 21*	88 ± 22*
Non-completers	777	79 ± 28*	85 ± 23*	84 ± 23*
**Lifestyle characteristics**				
**Physical Activity**	547			
Active	157	84 ± 24	89 ± 21	89 ± 20
Inactive	390	84 ± 24	88 ± 21	88 ± 22
**Nutrition *(Fruit and Vegetables)***	464			
Sufficient	71	88 ± 19	92 ± 15	93 ± 16*
Insufficient	393	84 ± 24	87 ± 22	87 ± 22*
**Nutrition *(Breakfast)***	545			
Regular	481	83 ± 25*	87 ± 21*	88 ± 21
Irregular	64	92 ± 17*	92 ± 20*	90 ± 22
**Smoking**	543			
Active smoking	49	87 ± 21	81 ± 29	80 ± 31
Passive smoking	62	84 ± 23	86 ± 23	86 ± 23
Non-smoking	432	84 ± 24	89 ± 20	89 ± 20
**Body Mass Index**	485			
Underweight	12	87 ± 21	93 ± 20	93 ± 19
Normal weight	321	83 ± 25	87 ± 22	87 ± 23
Overweight	101	85 ± 25	89 ± 20	90 ± 20
Obese	51	92 ± 15	93 ± 17	92 ± 19
**Number of Healthy Lifestyle characteristics**	401			
0	7	100 ± 0	100 ± 0	100 ± 0
1	31	87 ± 21	86 ± 25	85 ± 27
2	108	85 ± 24	86 ± 23	87 ± 24
3	153	83 ± 24	88 ± 22	88 ± 21
4	86	87 ± 21	92 ± 16	91 ± 18
5	16	83 ± 24	89 ± 18	94 ± 12

Note: These analyses only include women who opened three or more quizzes.

Abbreviations; n: number, SD: Standard Deviation.

^a^ Online questionnaire completers.

* Significant at p < 0.05.

## Discussion

The present study examined the reach of Hello World, the representativeness of its users for all pregnant women in the Netherlands, and the relationship between program engagement and the lifestyle characteristics of participants. Furthermore, it was explored whether the program content that participants chose to access was related to their lifestyle characteristics.

The study showed that Hello World reached approximately 4% of its target population and that low educated women (10%) and immigrants (22%) were underrepresented. On average, women received 6.1 quiz emails and opened 32% of the associated quizzes. A significant positive association was found between the number of received quizzes opened and the number of healthy lifestyle characteristics. After opening a quiz, women accessed the majority of questions on smoking (84%) nutrition (88%), and physical activity (88%).

Comparing the reach of Hello World to that of similar programs is hampered by the lack of other estimates of the actual reach of real-world (population-based) Internet interventions [13]. However, it is important to mention that the reach of Hello World was considerably lower than that of its previous version (4% versus 8%; [15]). It can be argued that this difference was caused by the fact that program reach was estimated somewhat differently in the previous study [15]. The previous study [15] used the number of living newborns as the denominator, whereas in the present study the number of living newborns and stillbirths was used. However, re-estimating the previous reach by including the estimated number of stillbirths (n = 781) [27] lead to a negligible difference. Furthermore, since Hello World has been available online since 2006, a proportion of potential program users may have already participated in the program during a previous pregnancy period and therefore, may have been less inclined to register for the program during the study period. Differences in recruitment strategies between the present and previous version of Hello World may also explain this finding. Hello World stopped using various traditional mass media channels (e.g. advertisements in magazines and an information stand at a national pregnancy fair). Instead, extra effort was made to recruit participants through midwife practices and gynaecologists by introducing the so-called “Hello World Newsletter” that was distributed in waiting rooms. A study evaluating the influence of various recruitment strategies on the reach of a web-based smoking cessation program supports this finding. More participants were recruited by using a mass media approach than through health professionals. The study did indicate, however, that recruiting participants through health care professionals was more successful in reaching people with low levels of education [28].

Although special efforts were made to reach immigrants and women with low levels of education, relatively few registered for the program. This was also the case for the previous version of Hello World and is in line with other research findings [12,15]. One study, for example, investigated predictors for visiting, using, and revisiting an online health communication program. They also found program users to be highly educated compared to the Dutch population at large, and most program users were native Dutch as well [12]. Research indicates that special recruitment strategies are needed to reach women with low levels of education and immigrants [28-31], but most of these strategies were not used by Hello World.

Relatively few program users reported complying with the Dutch Health Council fruit and vegetable recommendation (13%) and/or smoked during their pregnancy (12%) [7-9]. The percentage of program users who were overweight (22%) and obese (11%) before their pregnancy were in line with previous findings that indicating that approximately 23% of Dutch women of childbearing age are overweight and 10% are obese [10]. The percentage of participants who complied with the Dutch recommendation for physical activity was considerably lower than the percentage found among Dutch women of childbearing age (30% versus 50%) [10]. This might have been expected due to the physical changes and (partly inaccurate) risk perceptions about physical activity during pregnancy [32].

The number of quiz emails received did not differ between pregnant women with healthy and unhealthy lifestyle characteristics. However, engagement in more healthy behaviours was positively associated with opening more quizzes. This is in accordance with previous research findings [12,19]. One study, for example, found unhealthy habits for eating and physical activity at enrolment to be a predictor of non-usage attrition in a group of real-world participants to a web-based weight loss program [19]. This may be explained by the fact that people with unhealthy lifestyle characteristics are thought to be less motivated to pursue and maintain health and are therefore less inclined to actively engage in a health promotion program like Hello World [12,19].

After opening a quiz, women accessed the majority of questions on smoking (84%), nutrition (88%), and physical activity (88%). Significant relationships were found between several lifestyle characteristics and the percentage of smoking, physical activity, and nutrition questions accessed. However, between-group differences were relatively small, quiz topics were (by and large) unrelated to the lifestyle characteristics, and inconsistencies were found in the directions of these associations. Therefore, in contrast to our hypothesis, the program content that participants chose to access seems generally unrelated to their lifestyle characteristics.

### Practical implications

For the health information provided by Hello World to reach its target population, the greatest challenges seem to be increasing the reach of the program and to ensure that women with unhealthy lifestyle characteristics open a quiz.

Increasing the reach of Hello World may be accomplished by integrating the program in standard midwifery care [23] and/or increasing the awareness among pregnant women about the existence of Hello World by extending the use of mass media strategies. Recruitment of pregnant women with low levels of education and immigrants needs special attention, since tobacco exposure during pregnancy (both active and passive smoking) is found to be more prevalent among lower educated women [33,34] and overweight and obesity among lower educated women and immigrants [29,34]. Recruitment through health care professionals seems to be a useful strategy for reaching women with low levels of education [28]. Therefore, Hello World may put extra effort into recruiting these participants through midwife practices and gynaecologists situated in neighbourhoods with a lower socioeconomic status. Furthermore, although the health information is already presented in plain language and short text blocks, more visual aids (e.g. photographs, pictographs, and videos) and voice-recorded text messages could be incorporated in the program. To reach more immigrant women, another strategy should be considered. Previous research indicates that it is important to explicitly incorporate immigrants’ cultural beliefs, and those of the communities they represent, into the program and recruitment strategies. To accomplish this, it is essential to collaborate with key community members throughout the intervention design and implementation process [30,31,35], since this may foster shared ownership and assist with the identification of existing resources [35].

Hello World uses monthly email prompts to inform women about the availability of a new quiz, which is thought to be an effective strategy for improving program engagement [14]. However, as the use of *tailored* emails has also been found to increase program engagement [36]. The likelihood that women with an unhealthy lifestyle open a quiz might further be increased by personalising these emails to their health behaviour and/or (perceived) health status. Furthermore, since women with an unhealthy lifestyle are thought to be less motivated to pursue and maintain their health [12,19], strategies other than alerting them about the availability of new health information should probably be used as well. For example, content relating to their interests, but not necessarily health, could be incorporated in the program [19]. Another strategy to increase their program engagement may be the introduction of incentives; the number of correct answers can be tracked and small incentives could be offered if a pre-set percentage of all quiz questions is answered correctly.

### Strengths & limitations

Strengths of the present study are that it is one of the first systematic studies on the reach and use of Internet-based interventions, its population-based design, and the objective registration of program use. Furthermore, we were able to investigate whether accessing program content about a specific lifestyle topic was related to the lifestyle characteristics of the participants. Since many interventions aim to achieve health behaviour change by simultaneously providing information on various lifestyle topics, the finding that participants accessed health information largely independent of their lifestyle characteristics may be of importance for the general field of preventive medicine. However, further research is needed to confirm this finding in other populations and to investigate the strategies that might be used to increase the likelihood that information about a specific lifestyle topic reaches those who may benefit most from it.

Several limitations to the present study are noteworthy. First, the response rate to the lifestyle questionnaire was relatively low (31%) and completers and non-completers differed in terms of their level of program engagement. However, since the lifestyle characteristics of the completers were by large in accordance with those found in Dutch (pregnant) women and completers and non-completers did not differ in terms of their education level, a strong predictor of an individual’s health status [37], this probably did not distort our conclusions. Second, to estimate the reach of Hello World, the number of miscarriages in the Netherlands during the 7-month study period was not taken into account. This may have resulted in an overestimation of the program reach. However, since women were on average 15 weeks pregnant at registration and most miscarriages take place during the first trimester of pregnancy (1–12 weeks), the number of miscarriages was probably small. Furthermore, as previously pointed out [15], a drawback of calculating program reach based on the number of participants who register for a program is that women who were not exposed to the intervention were also taken into account. This should be kept in mind while interpreting the results and comparing them to other studies.

## Conclusions

Between March 25, 2009 and October 25, 2009 Hello World reached fewer pregnant women compared to the previous version of the program that was available online between November 13, 2006 and November 13, 2007. The number of quiz emails received did not differ between women with specific healthy and unhealthy lifestyle characteristics, but active participation in the program was positively associated with the number of healthy behaviours participants engaged in. Furthermore, the program content that participants chose to access was largely unrelated to their lifestyle characteristics.

## Competing interests

The authors declare that they have no competing interests.

## Authors’ contributions

JvD, MvP, IM and JB contributed to protocol design, data analyses and data interpretation. JvD wrote the manuscript and received comments from MvP, IM, JvO, JB. All authors have seen and approved the final version of this paper.
